# Uncovering Burdens, Examining Needs, and Shedding Assumptions of Evidence-Based Social Support Programs for Mothers: A Descriptive Qualitative Study in a Remote Community

**DOI:** 10.1177/23333936211035747

**Published:** 2021-07-31

**Authors:** Johanna R. Jahnke, Julee Waldrop, Alasia Ledford, Beatriz Martinez

**Affiliations:** 1 University of North Carolina at Chapel Hill, USA

**Keywords:** stress, distress, interventions, support groups, caregivers, caretaking, internet, technology, remote, rural, health care, social support, community and public health

## Abstract

Many studies have demonstrated a significant burden of maternal stress and depression for women living on the Galápagos Islands. Here, we aim to uncover burdens and needs of women with young children on San Cristóbal Island and then explore options for implementing evidence-based programs of social support to meet these needs. We conducted 17 semi-structured qualitative interviews with mothers of young children, healthcare workers, and community stakeholders. We then used Summary Oral Reflective Analysis (SORA), an interactive methodology, for qualitative analysis. Despite initial reports of a low-stress environment, women described many sources of stress and concerns for their own and their children’s health and well-being. We uncovered three broad areas of need for mothers of young children: (1) the need for information and services, (2) the need for trust, and (3) the need for space. In response to these concerns, mothers, healthcare workers, and community leaders overwhelmingly agreed that a social support program would be beneficial for the health of mothers and young children. Still, they expressed concern over the feasibility of such a program. To address these feasibility concerns, we propose that a web-based education and social support intervention led by nurses would best meet mothers’ needs. Women could learn about child health and development, develop strong, trusting friendships with other mothers, and have their own space to speak freely among experts and peers.

## Introduction

Over the last decade, maternal mental health has become a growing concern for the World Health Organization and other governing health agencies. Depression, in particular, is the leading cause of disease burden for women worldwide (Albert, 2015). In addition to the social, emotional, and economic consequences of compromised maternal mental health, children of depressed mothers are at risk for poorer developmental and behavioral outcomes and poorer health overall (Atif et al., 2015; Surkan et al., 2011). Maternal distress, including maternal stress, depression, and anxiety, can shape a child’s behavioral and physiological development (Dunkel Schetter & Tanner, 2012). Rates of depression are estimated to be two to three times higher in low- and middle-income countries than high-income countries (Fisher et al., 2012). In particular, on the Galápagos Islands, studies have demonstrated a significant burden of both stress and depression on women (Jahnke, Terán, et al., 2021; Page et al., 2013; Thompson et al., 2020).

Here, we engage the cultural safety (Ramsden, 1993) approach to follow up on our previous work with women on San Cristóbal Island to uncover the burdens and needs of women with young children and to explore options for implementing evidence-based programs of social support to address these needs. First, we offer a brief background on the people of the Galápagos, the role of social support in reducing symptoms of stress and anxiety, and our methods for accomplishing the aims of our study. We then present our findings related to our study aims: *uncovering burdens, uncovering needs*, and *exploring solutions*. Finally, we discuss the implications of our results as they relate to both future research and nursing practice. In observing the cultural safety approach in research, we strive to acknowledge our positionality, be conscious of power dynamics, and benefit the community (Mkandawire-Valhmu, 2009, 2018).

### The People of The Galápagos

The Galápagos Islands are located approximately 600 miles off the west coast of Ecuador in the Pacific Ocean. While they are most well-known for their unique sea and terrestrial life, the islands are home to 25,000 residents on four islands: Santa Cruz, San Cristóbal, Isabela, and Floreana (INEC, 2015). As the Galápagos does not have an indigenous population, its human population remained minimal before the mid-20th century. Since the 1960s, the creation of the Galápagos National Park in 1959 and the designation of the Galápagos as a United Nations Educational, Scientific and Cultural Organization (UNESCO) World Heritage Site in 1978 incited population growth, and tourism and migration brought with it economic and urban development (Hoyman & McCall, 2012; Walsh & Mena, 2013). Now, of the 7,200 residents of San Cristóbal Island, nearly all (~6,500) live in the province’s capital city, Puerto Baquerizo Moreno, while the others reside in the highlands of El Progreso (INEC, 2015).

Factors that may contribute to stress and depression in mothers of young children in the Galápagos include concerns about access to high quality and specialty health services (Jahnke, Archer, et al., in press; Page et al., 2013), limited access to clean water (Gerhard et al., 2017; Houck et al., 2020; Nicholas et al., 2020), the availability of nutritious foods (Freire et al., 2018; Page et al., 2013; Pera et al., 2019; Thompson et al., 2020), exposure to intimate partner violence (INEC, 2011; Villacis & Carrillo, 2013), and the financial demands of a tourist economy. Further, many women on the Galápagos have migrated from mainland Ecuador. Migrants also have higher rates of depression than their non-migrant counterparts (Fellmeth et al., 2017).

Ecuador’s healthcare system has been restructured over the last few decades, transitioning from primarily private to increasingly public care (De Paepe et al., 2012; Rasch & Bywater, 2014). As a piece of this movement, in 2008, Ecuador adopted a new constitution ensuring health care is a right and guaranteeing free care to all citizens (Aldulaimi & Mora, 2017; De Paepe et al., 2012). Public health care now accounts for most health facilities in Ecuador (Pan American Health Organization [PAHO], 2008), and it is often the only option in more rural areas. However, private facilities remain throughout the country for those who choose it and can afford it (López-Cevallos & Chi, 2010). After adopting the new constitution, Ecuador entered into bilateral cooperation agreements on health with Cuba through which Ecuador has invited Cuban physicians to work throughout Ecuador (Anderson, 2015), including in the Galápagos. These physicians have been met with some resistance from the Galapaganean population, who preferred Ecuadorian physicians (Jahnke, Archer, et al., 2021). Though bilateral cooperation agreements dissolved at the end of 2019, they were still in effect when this research was conducted. Over the past decade, amid the significant shifts in health care provision, Ecuadorians have reported being disappointed in public health care (De Paepe et al., 2012; Rasch & Bywater, 2014), which has influenced its utilization and ability to improve health among Ecuadorians (Adane et al., 2017).

While free public health services are available at all the island’s health centers, including a hospital and a comprehensive primary care center in Puerto Baquerizo Moreno and a community health care center in the highlands, mental health care is limited. Further, privacy concerns have deterred some residents from utilizing this care to its fullest extent (Waldrop et al., in process). These concerns may stem from the fact that residents are still building trust with the island’s new hospital, which opened in 2016. Since health care providers have work contracts for only a few years at a time (Jahnke, Archer, et al., 2021), the community has not had the opportunity to build long-term trust with individual providers, making intimate conversations about mental health and depression difficult. Further, island residents feel that patient confidentiality is not always upheld on the small island, preventing patients from seeking the care they may need (Waldrop et al., in press).

### Social Support

Social support is associated with lower stress (Raffaelli et al., 2013), depression (Dennis, 2003; Razurel et al., 2013; Robertson et al., 2004), and anxiety symptoms (Glazier et al., 2004) in many settings, and it may be an effective tool for improving maternal mental health on the Galápagos Islands (Fellmeth et al., 2017). Social support includes emotional support (expressions of caring, empathy, and trust), informational support (advice or guidance), affirmational support (validation of feelings and experiences), and instrumental support (physical or practical assistance with tasks or material aid) (Dennis, 2003). Emotional support, which is founded on trust, promotes feelings of safety and belonging (Bäckström et al., 2017). Research has found that emotional support is essential for a positive experience of support, which is necessary to mitigate the consequences of stress (Bäckström et al., 2017). Emotional and affirmational social support may increase a woman’s confidence in coping with stress, allowing her to respond in a more physiologically well-regulated way and thus avoid chronic stress that could be detrimental to health (Jewell et al., 2015; Uchino, 2006). Instrumental social support can provide women with trusted allies with whom they can leave their child(ren) to complete other tasks, sleep, or just regroup and rest. Social support is crucial for new mothers to protect maternal health and infant development (Jewell et al., 2015). Further, women with more informational social support have demonstrated differences in parenting behaviors (Bäckström et al., 2017; Green et al., 2007).

However, in the Galápagos, community-based programming that might provide social support and mental health symptom relief to mothers with young children is limited. In 2018, *Establecimientos Amigos de la Madre y del Niño (ESAMyN)*, a hospital-based program designed to teach pregnant women about pregnancy, childbirth, and infant development, was rolled out as a part of Ecuador’s broader government effort to improve health care services across the country (Ministry of Public Health [Ecuador], 2020). While this program was led by nurses and designed to teach women about maternal and infant health, it also built stronger relationships among residents and hospital staff. Additionally, it provided a prolonged support system for women during their pregnancies. Women used the sessions to garner informational support from staff and seek emotional and affirmational support for their parenting and mental health concerns. Despite the program’s popularity among women on the island, *ESAMyN* was discontinued within the year due to staffing and resource shortages. On the island of San Cristóbal, now only one community support program for women exists. This program focuses on breastfeeding awareness and education, not broader themes of motherhood, social support, or mental health.

## Methods

### Study Design

This study employed qualitative descriptive methods (Sandelowski, 2000) using semi-structured interviews to allow maximum content to emerge from participants. We developed guiding questions based on our previous research experience in the Galápagos and meetings with people from the Galápagos and other experts in the field. Specifically, we consulted experts in public health, anthropology, and geography and examined historical conversations with residents of the Galápagos.

### Researcher Positionality

All research team members are women, are fluent Spanish speakers, and are part of an interdisciplinary group involved with the community, the Center for Galápagos Studies (CGS). One author (Waldrop) has been involved in maternal and child health research in the Galápagos for over 10 years. Another author (Jahnke) has been involved in maternal and child health research on San Cristóbal for 5 years. During this time, she lived on San Cristóbal for 1 year while collecting data for a separate research project on maternal mental health and subsequent infant health before the development of this study. The research team developed this project out of a desire to use previous research to inform and further distill the needs of mothers on the Galápagos and ultimately serve the community by helping to address these needs. We recognize that our positionality as outsiders (academics and non-Ecuadorians) influences this work’s results. Participants may be reticent to acknowledge systemic problems on the islands, knowing that the CGS partners with the government and the local health system for other projects. Power dynamics stemming from our outsider status may shape both women’s willingness to participate in the study and their responses to interview questions (Mkandawire-Valhmu et al., 2009). Nonetheless, our lasting relationship with the community through the CGS has built trust over 10 years. Residents are aware of the center’s longstanding commitment to both scientific and community projects.

### Data Collection

We collected data in June 2019. We recruited participants from known past research participants and referrals they made to the researchers using snowball sampling techniques. All recruitment was done in person or by phone. We used purposive sampling to ensure that mothers of young children, healthcare workers, and community leaders were well represented, as they may play a future role in supporting, organizing, or providing social support services. Inclusion criteria for mothers of young children required that they be full-time residents in the Galápagos and have a child under 3 years of age. Inclusion criteria for healthcare workers, doctors, or nurses required that they were currently employed at the local hospital. Inclusion criteria for community leaders required that they were full-time residents of the Galápagos and that they either currently or previously worked in community organizations, local government, or local business efforts on the island.

We conducted 17 semi-structured qualitative interviews. We first inquired about the challenges that mothers of young children face, and second, requested feedback on various programming strategies designed to meet these needs. The interview guides were worded differently depending on the participant. For example, mothers were asked, “Others have told us that sometimes it is stressful to live here. Do you agree with that statement or not and why?” In contrast, healthcare workers or community leaders were asked, “What do you feel are the biggest stressors for mothers with young children (0-3) on the island?” Similarly, we asked mothers, “Here are some examples of things that some women find helpful to learn about and talk about . . . What are your thoughts on these topics?” The others were asked, “Do you feel that women with young children have unmet needs in any of the following areas. . . . How do women cope with these things?” While we asked all participants similar initial questions, later questions evolved from participant responses throughout each interview.

Of the 17 interviews conducted, eight were with mothers of young children, six were with healthcare workers, and three were with other community leaders. Qualitative work does not lend itself to exact sample size; the research team evaluated the adequacy and comprehensiveness of interviews to ensure data saturation was met (Morse, 1995).

We conducted all visits in Spanish in the participants’ homes, places of work, or the local hospital according to participant preference. All participants provided written informed consent before participation. We advised all participants of the voluntary nature of their involvement, including the right not to answer any question and their ability to terminate the interview at any time. Upon completing the interview, we compensated participants $5.00 U.S. dollars (the currency in Ecuador) after careful consideration of the average value of their time (Mkandawire-Valhmu et al., 2009). The interviews were recorded and deposited into a password and firewall-protected university-approved data and documented online storage system. The Institutional Review Boards for the University of North Carolina at Chapel Hill and *Universidad San Francisco de Quito* approved this study.

### Data Analysis

We utilized Summary Oral Reflective Analysis (SORA) to analyze all qualitative descriptive data (Thompson & Barrett, 1997). This approach, developed by nurses, utilizes an interactive method that seeks to preserve the contextuality and richness of data and calls upon the researcher to actively and orally reflect on concepts, meanings, and themes emerging from the data (Thompson & Barrett, 1997). While other qualitative analysis methods rely on reading participants’ literal transcripts, inherently precluding contextuality and striving for unattainable objectivity, SORA recognizes the need to actively listen to the participant’s voice and reflect on the contextuality. This approach facilitates hearing the data speak, allowing the researcher to remain close to the data so that central themes emerge with greater clarity. Interviews with participants were audio-recorded and transcribed verbatim in Spanish by an encrypted, secure transcription service with a non-disclosure agreement (www.vananservices.com). All members of the research team are fluent in Spanish. According to SORA protocol, the team simultaneously and collaboratively listened to the recorded interviews while reviewing the transcripts. The team members then provided their oral reflections around themes, ideas, and conceptual linkages. These were also audio-recorded, transcribed, and used in subsequent analysis. Throughout this process we returned to the original audio-recordings to clarify meaning, check potential researcher bias, refine themes, and extract representative quotes.

### Strategies to Ensure Trustworthiness

This methodological approach of oral reflection, recording and revisiting the reflection, and returning to the participant data as a research team assisted in ensuring rigor and trustworthiness. Investigator triangulation (Sandelowski, 1993) involved four researchers during the analysis. Additionally, the methodological approach of SORA assisted researchers in identifying and clarifying potential researcher bias (Morse, 2015). For example, when participants discussed the cultural expectations of women and the “machista” attitudes within the community, the methodological approach of SORA required researchers to discuss and orally reflect on whether the derived themes related to these comments reflected the voice of the participants or the researchers. Disconfirming evidence, evidence from the data demonstrating that a working theme or theory is incorrect (Lincoln & Guba, 1985), was evaluated by comparing derived themes within groups (mothers, healthcare workers, and leaders) between groups. For example, participants’ initial denial of stress on the island presented as disconfirming evidence. Researchers compared this finding both within and across groups to better understand the burdens experienced by participants. After refining themes, the research team performed Synthesized Member Checking (Birt et al., 2016) with a sample of participants via WhatsApp Messenger, an internet-based messaging application for smartphones, to confirm conclusions and ensure the reliability of findings. Because our focus was on mothers’ needs, through this process, we sent an outline of the key themes we identified to four of the eight participating mothers individually and asked for reactions and feedback. In response, the mothers overwhelmingly agreed with our findings, and each emphasized particular results that were most relevant to them.

## Results

### Uncovering Burdens

Interviews with mothers of young children demonstrated that women ultimately detail a litany of concerns and needs despite initial reports of a tranquil and easy lifestyle on the islands. Initially, when asked directly about their burden of stress in interviews, most women denied any feelings of stress. In response, women often pointed to their good fortune to live on the Galápagos, which is quiet, peaceful, and connected to nature compared to mainland Ecuador, which many participants described as loud, crime-ridden, and dangerous. When first questioned about the stress in their lives, mothers of young children responded:

*A ver, diría que no es tan estresante porque aquí es más tranquilo, porque no hay mucha bulla de carros, no hay muchas personas. Es un lugar muy lindo. No, no es tan estresante.*

Let’s see; I’d say it isn’t as stressful here [in the Galápagos] because it’s calmer, because there isn’t a lot of noise from cars; there aren’t a lot of people. It’s a very beautiful place. No, no, it isn’t so stressful.

(Mother, 30–40 years old)

*No hay estrés en Galápagos. Porque no hay mucho tráfico, no hay violencia, no hay. . . y el tiempo como que va más despacio también que en las grandes ciudades.*

There is no stress in the Galápagos. Because there isn’t much traffic; there isn’t violence, there isn’t . . . and time—it goes slower also than in the big cities.

(Mother, 30–40 years old)

Despite initial reports of a low-stress life, throughout interviews, women described many sources of stress and concerns for their own and their children’s well-being, all the while maintaining the mantra of living in paradise. In addition to describing their anxieties around previously reported issues for residents of the Galápagos, including food shortages, quality, and diversity of food; machismo; and financial insecurity, women discussed additional burdens, which we categorized into three areas of need for mothers of young children: (1) the need for trust, (2) the need for information and services, and (3) the need for space.

### Uncovering Needs

#### Need for trust

In interviews, mothers of young children repeatedly discussed their mistrust of others on the island. This mistrust prevented most women from feeling that they could be vulnerable enough to form close friendships. Most women suggested that their support system consisted exclusively of family, particularly the woman’s family, even if they lived on the mainland. Others, however, and particularly those who did not have a close relationship with their local families, reported having no social support at all. One participant said:

*No sé, me siento sola, no tengo con quien hablar, cada vez la preocupación se aumenta más, y a veces me llega la desesperación, y digo: “¿qué voy a hacer o qué irá a pasar?”*

I don’t know, I feel alone; I don’t have anyone with whom I can talk; each time, my worry increases, and sometimes I become desperate, and I say, “What am I going to do, or what’s going to happen?”

(Mother, 20–30 years old)

Another woman, who reported having friends on the island, described only tenuous feelings of trust and connection. When asked if she felt she had her friends’ support in helping her with children, she replied:

*No le vas a decir a la amiga: “Ven, cuídame al bebé”*

You’re not going to say to a friend, “Come and watch the baby.”

(Mother, 30–40 years old)

This response and others demonstrated discomfort and mistrust in relying on friendships for support. Further, others’ responses suggested that it is a fear of community gossip that prevents residents from forming close connections with others on the small island. For example, one woman reported:

*Se siente mal porque la isla es pequeña, entonces cualquier cosa que pase, lo saben. Pero no es que lo saben de buena manera. Lo saben pero solo para murmurar, no para ayudar.*

It feels bad because the island is small, so then, whatever happens, they [Galápagos residents] know. But it’s not that they know about it in a good way. They know it but just to gossip about it and not to help.

(Mother, 20–30 years old)

Another central area of social mistrust stems from the residents’ poor experiences with healthcare providers on the island. Often, women reported that this resource does not fulfill its obligations to the community, especially for mothers with young children. Women often discussed disagreeing with or not understanding the feedback they receive from health care providers on the island, demonstrating a disconnect in health literacy between patients and providers and perpetuating mistrust between the two groups. Resentment between these groups has also grown from the residents’ perception that healthcare providers do not seem busy but leave patients waiting for care. They feel that providers do not always uphold standards of privacy and confidentiality, exacerbating issues of trust both within the health care system and within the community where the health care workers reside.

The healthcare workers were also aware of the residents’ mistrust. They expressed frustration when discussing resident interaction and adherence to treatment and recommendations. For example:

*Veo que preguntan mucho sobre el tratamiento que estamos dando, pero siempre noto como que hay un poco de desconfianza, y por eso es que lo veo defensivo con respecto al trato.*

I see that they [patients] ask a lot about the treatment we [providers] are giving, but I always notice that there is a bit of mistrust, and that is why I see it as defensive regarding the treatment.

(Healthcare worker)

*También la gente tiene mucha desconfianza en los profesionales porque son rurales y porque son extranjeros.*

Also, the people have a lot of mistrust in the professionals because they [the people] are rural and because they [providers] are foreigners.

(Healthcare worker)

Almost all the healthcare providers on the island were from the mainland or other countries. They received training in traditional Western medicine culture, which is mainly patriarchal and includes a firm belief that “my way” is the right way. One healthcare worker described:

*Ellas creen al momento que con algún antibiótico ya deben curarse o ya deben tener mejoría. Y tienen poca expectativa con respecto al tratamiento que se les da. Entonces siempre andan buscando una segunda opinión, o una tercera, para quedar así conformes.*

They [patients] believe at the moment that with some antibiotics, they should already be cured, or they should already have improvement. And they have little expectation regarding the treatment given to them. So, they are always looking for a second opinion, or a third, to be satisfied.

(Healthcare worker)

#### Need for information and services

Interviews with mothers revealed that many resources are perceived as unavailable, inaccessible, or inferior to services on the Ecuadorian mainland. In particular, women spoke of their concerns about health care services and education, and particularly their children’s physical, social, and emotional development on the islands. Mothers of young children reported:

*A veces no hay medicamentos. Por ejemplo, hay ciertos medicamentos que no los venden aquí.*

Sometimes there isn’t medicine. For example, there are certain medicines that they don’t sell here [Galápagos].

(Mother, 30–40 years old)

*. . . en estos dos años todavía no habla y . . . sí, a veces me preocupo porque tanto y tanto trabajo, no enseña a hablar bien, no sé qué es lo que pasa. En eso me preocupo.*

. . . in these two years, he [my son] still doesn’t speak and yes, sometimes I’m worried because I work and work so much, he isn’t showing to speak well; I don’t know what’s happened. I worry about that.

(Mother, 20–30 years old)

*Aquí en la isla no hay [psicólogos]. Por ejemplo, yo cuando estuve mal, triste, mi mamá me consiguió una cita pero en la parte continental, en Guayaquil. Pero aquí, no.*

Here on the island, there aren’t [psychologists]. For example, when I was doing badly, sad, my mother got me an appointment on the mainland in Guayaquil. But here, no.

(Mother, 20–30 years old)

*Yo lo llevé a donde una especialista porque me preocupé mucho, porque un día, me hizo un berrinche muy grande que se aguantó todo el oxígeno y se puso morado, y yo dije: “ya me estoy saliendo de control”, porque es como que una rabieta que yo lo vi que se estaba desmayando de lo que hizo. Entonces yo quería controlarlo para ver qué estaba pasando, si es que él tiene algo, o si es que pasa algo con él realmente. Por eso lo llevé ahí [el continente]. Es un poco costoso. Pero tenía que hacerlo porque tengo que estar segura que mi hijo esté bien. Y no todas las madres lo pueden hacer.*

I took him to a specialist because I was very worried, because one day he threw a very big tantrum that took all the oxygen, and he turned purple, and I said, “I’m already losing control” because it’s like the tantrum that I saw . . . he was fainting from what he did.

I wanted to monitor him to see what was really going on, if he had something or if something was really wrong with him. That’s why I took him there [to the mainland]. It’s a little expensive. But I had to do it because I have to make sure my son is okay. And not all mothers can do that.

(Mother, 20–30 years old)

The healthcare workers and community leaders also expressed an understanding of the needs on the islands, such as the lack of early (before kindergarten) and higher (beyond high school) education and low health literacy. Healthcare workers also felt frustration and a sense of fatalism with the community members, whom they perceived as not following their advice and seeking other care sources. Ultimately, a disconnect emerged between healthcare workers’ perceived frustration of residents and their perception that they had no responsibility for the status quo because they fulfilled their obligation to patients by telling them what they should do for their health problems. Speaking about the island residents, a healthcare worker reported:

*Generalmente, son pacientes que no todos tienen una buena formación, no todos alcanzan los estudios universitarios, no todos alcanzan los estudios secundarios, mucho menos*.

Generally, they [Galapagos residents] are patients that don’t all have a good education, not all reach university studies, not all reach high school education, much less.

(Healthcare worker)

A community leader echoed this sentiment:

*No son fuertes económicamente, ni emocional, ni mental en nada.*

They [Galapagos residents] are not strong economically, emotionally, or mentally at all.

(Community leader)

Both participants demonstrate an awareness of the needs of community members yet maintain distance and separate themselves from that need, with the phrases “They are patients that don’t all have a good education” and “They are not strong . . . .” Thus, the disconnect persists between the perceived need and the acknowledgment of their responsibility to better provide the community support to meet that need.

#### Need for space

Last, interviews with mothers of young children revealed their need for physical and emotional space to improve their own and their children’s health and well-being.

Women often spoke of their struggles with physical space, describing how small the island is and the fact that the community is always watching, harkening back to concerns of gossip and mistrust within the community. Further, though the island of San Cristóbal is large, 97% of the geographic area of the Galápagos is designated to the Galápagos National Park (Walsh & Mena, 2016), much of which is inaccessible without a park guide and associated fees. Thus, while many women report that life in the Galápagos is a natural paradise, their descriptions of daily life suggest that much of nature is inaccessible or limited by a steep cost.

Even aside from natural spaces, women discuss feeling physically limited within their small city. Mothers often reported strong machista sentiments in the community that emphasize different standards of acceptable behavior for women and men. The culture normalizes men’s freedom to live an independent life. At the same time, women feel confined to the home where they are expected to complete most, if not all, household and childcare responsibilities. Many women reported feeling overwhelmed by this expectation. They struggled to manage their health, their children’s health, schoolwork, cooking, chores, and relationships with their partners. Community leaders and healthcare workers reported:

*Es como que hablan del cansancio, de la falta de apoyo del marido. Muchas veces no hay mucho apoyo por parte del marido. Todavía somos una sociedad bastante machista.*

It is like they [mothers] talk about fatigue, the lack of support from the husband. Many times, there is not much support from the husband. We are still quite a “machista” society.

(Community leader)

*Pero no le da ni un solo momento para que ella pueda salir a recrearse, no le da ese espacio para que ella pueda salir a la playa o a caminar o a hacer ejercicio. No. Simplemente le dice: “estás gorda y necesito que me prepares la comida.” Y ella también trabaja. O sea, ella también trabaja, ella también está estudiando en línea.*

But he [a husband] doesn’t even give her a single moment for her [wife] to relax; he doesn’t give her the space to be able to leave or go to the beach or walk or do exercise. No. He simply says, “you’re fat, and I need you to prepare my meal.” And she also works. As such, she also works, and she is also studying online.

(Community leader)

*El padre pues es más como que . . . todo haga la esposa, que todo haga la mujer. La crianza de los hijos para la mujer, la lactancia, el cuidado, la educación, entonces él provee en algunos de los casos, no siempre.*

The father is well, more like . . . that the wife does everything, that the woman does everything. Raising the children is for the woman, breastfeeding, caring for, education, so he provides in some cases but not always.

(Healthcare worker)

*Porque bueno, aquí en Galápagos al menos se ve mucho el machismo. Y eso es uno de los factores por los conflictos de las parejas.* (Community leader)

Well, here in Galapagos, at least you see a lot of machismo. And that is one of the factors for the conflicts of the couples.

(Community leader)

Women’s confinement to the home space is further exacerbated by the islands’ job market, as men often work in the fishing or tourism industries, both of which require long hours and even days and weeks away from home. Women’s limited physical spaces, in turn, seclude them from the community and prevent them from the time and space necessary outside of the home to forge strong friendships, contributing to the insular stronghold of the family and limiting community ties for women.

Women also describe the need for a safe, emotional space to think, reflect, and connect. In interviews, women repeatedly suggested that it would be “risky” or “unsafe” to discuss concerns about their home life to either peers or health care providers due to the potential consequences of gossip. Many women, though, did desire safe, trusted places where they could openly discuss their concerns. One woman suggested that they needed a place where they could discuss the concerns of mothers, women, and children to build each other up rather than gossip about each other behind their backs. Currently, there is a breastfeeding group for new mothers that meets bi-weekly at the local hospital. One participant praised this group, saying:

*Entonces interactuar con las madres y poder preguntar o escuchar cosas que ellas ya han vivido, así. Entonces yo pienso que está bien porque uno, cuando está pasando algo con su bebé, piensa que ella solo lo pasa. Entonces hay más madres y familias que están pasando por lo mismo.*

So to interact with the mothers and be able to ask or listen to things they already have experienced, like that. So, I think that this is good because when one is going through something with her baby, she thinks it is only happening to her. So, there are more mothers and families that are going through the same thing.

(Mother, 30–40 years old)

### Exploring Solutions: Feedback on Programming Suggestions

When we asked mothers, healthcare workers, and community leaders if they thought some type of educational or social support program would be beneficial for women with young children, they all agreed it would. Still, they also agreed that there were many concerns to women’s participation, primarily logistics and trust.

In terms of logistics, both mothers and community leaders expressed concerns about scheduling meetings, meeting locations, group facilitators, and creating a safe space for children of different ages. Concerning scheduling a support group, one mother quickly pointed out that:

*La mayoría trabaja. Yo creo que los domingos porque el domingo creo que la mayoría tiene libre.*

The majority [of women] work; I think Sunday because Sundays, I believe, most have free.

(Mother, 20–30 years old)

When asked about locations and group facilitators, issues of trust emerged as essential considerations. As described by two mothers:

*. . . que el lugar esté bien adecuado para que estemos cómodas.*

. . . the place needs to be suitable so that we feel comfortable in it.

(Mother, 20–30 years old)

. . . para el encuentro así de madres sería un espacio como que un poco privado

. . . for a meeting like this of mothers, it would need to be a space that is a little private.

(Mother, 20–30 years old)

Community leaders echoed this sentiment. One community leader mentioned the possibility of meeting in a public space and then, after some thought, added:

*Sería bueno, pero aquí la gente es muy chismosa. . . .es como que vivimos en un lugar tan pequeño que todo el mundo se conoce, todo el mundo se sabe, y eso solamente sería como hacer leña del árbol caído.*

It would be nice, but here [Galápagos], the people are gossips . . . it’s like we live in such a small place that everyone knows each other, everyone knows, and this [approach] would be like adding wood to the fire.

(Community leader)

Concerning a group facilitator, many women suggested a professional, but one mother pointed out:

*Yo creo que puede ser alguien de aquí mismo, que tenga ese modo de llamar a la gente . . . buscar a la persona correcta es importante*

I think it could be someone from here, that has a certain way with the people . . . finding the right person is important.

(Mother, 20–30 years old)

Other participants raised concerns about the feasibility of arranging and paying for childcare during meetings and the cost of travel. In response, some suggested utilizing virtual spaces to meet these needs, creating groups within messaging apps like WhatsApp, which is used widely for communication and group organizations on the islands:

*Yo creo que el uno a uno y el WhatsApp. A la gente le encanta estar metida en el teléfono . . .*

I think one on one and WhatsApp. The people love to be on their phone.

(Healthcare worker)

Another community leader observed about Facebook:

*Yo pienso que de esa manera llegaría de una manera más rápida, porque todo el mundo ve. Si van a buscar algo, es por ahí.*

I think that this way [information] would be much faster because everyone would see. If they’re going to search for something, it’s there.

(Healthcare worker)

## Discussion

Overall, our results demonstrate a paradox in which women do not initially disclose concerns about their lives. Still, in-depth interviews reveal that lack of information and services, distrust, and limited spaces burden mothers of young children on the Galápagos Islands. In addition to these concerns, mothers must manage both work and households, which places them under tremendous stress. Stress is exacerbated when mothers seek specialty services for their children at the local hospital, where most of the workforce are transient health workers, and the island’s legacy of poor services has historically fostered mistrust for residents. In response to these concerns, mothers, healthcare workers, and community leaders overwhelmingly agreed that a social support program would be beneficial for the health of mothers and young children.

Motivated by the cultural safety framework, which encourages an iterative research process that ultimately assists in addressing the needs of the community (Mkandawire-Valhmu et al., 2009), the aim of this project was to draw on our previous research in the Galápagos to explore options for addressing the mental health needs of mothers. As a consequence, the discussion will center around considerations for implementing evidence-based programs of social support in alignment with the community’s goals.

### Considerations for a Social Support Intervention

Despite evidence that group social support has been effective in high, middle, and low-income countries (Rahman et al., 2013), many participants expressed concerns about the feasibility of implementing group social support programming on the island. Concerns about the feasibility of implementing such a program were primarily of mistrust and logistics.

In response to the suggestion of group-level programming designed to build relationships with other women, participants often expressed fear that group judgment and gossip could detract from the potential benefits of a support program. For example, the discontinued government-run *ESAMyN* education program was initially designed to be a group program that included social support. However, it was changed to an individual-level educational intervention during rollout because women felt uncomfortable sharing their health and pregnancy concerns with peers. While community trust poses one challenge, trust of healthcare providers poses another. Recurring themes of mistrust of healthcare providers’ medical capabilities and their commitment to upholding confidentiality and privacy suggest that careful consideration is needed in selecting a group leader who is an expert and an ally to community members.

Logistics pose a second potential challenge to social support programming. While we had initially envisioned an intervention that could provide social support through group meetings, this approach may not suit this setting. Traditional group programming faces many challenges in the context of the Galápagos. To hold a support group in person, a safe and neutral space where women would be comfortable convening and bringing their children is required. However, the financial realities faced by many women in the Galápagos must also be considered and may hinder the success of an in-person program. Women often expressed concerns about securing (and paying for) childcare for older children during group meetings. In addition, questions were raised about the optimal timing of the group and suggestions of groups meeting based on children’s age so that lessons could be tailored by age group and so that older children would not interfere with the play of younger children during group. Perfecting the timing of the meetings would also be essential for the success of an in-person program, as the group would need to complement existing schedules on the island, including those for children’s school, women’s work, and women’s obligations at home. The community’s limited resources (both nurses and participants) pose challenges for effectively formatting an in-person meeting that would benefit most women without excluding those who are particularly vulnerable.

### Nursing Implications

Given these context-specific concerns, along with the new unforeseen barrier of COVID-19, we propose the feasibility of an online social support program. Nurses, whose medical capabilities are less often called into question on the Galápagos, and whose expertise in maternal and child health may motivate participation from the community are the proposed leaders. The initial success of *ESAMyN*, which was organized and led by nurses, inspires confidence that nurses would be the best leaders for our proposed program as well. Throughout the duration of *ESAMyN*’s programming, the nurses were trusted, and community members were excited to receive advice from an approachable medical professional. While community-based health workers might be acceptable, the most critical aspects of successful interventions are local, trustworthy, and intersecting with the healthcare system (Gilmore & McAuliffe, 2013).

Although all types of social support could benefit mothers of young children in the Galápagos, informational and emotional/affirmational may be the best combination for this context (Fellmeth et al., 2017; Green et al., 2007). A recent systematic review of perinatal mental disorders in women in low and middle income countries reported that using both trained and supervised health workers in culturally appropriate community settings and focusing on problem-solving and psychoeducation are the most effective for improving maternal mental health (Rahman et al., 2013). This type of intervention, led or facilitated by nurses, could provide information derived from evidence and experts. Nurses would be the ideal experts for psychoeducation, coping strategies, parenting, and child development. An intervention of this nature, guided by nurses, would ideally offer solutions to the needs of mothers by building trust among community members and healthcare workers, providing information and services on maternal and child health and ultimately allowing a safe space for women to speak freely among their peers, develop trust, and strengthen friendships. Evidence-based programs that support parenting and child development, which were developed by and in conjunction with nurse researchers and interventionists, such as Alumbrando El Camino (Lighting the Way) (Beeber et al., 2011; Canuso & Beeber, 2009) and Baby Cues (NCAST, 2011), could be adapted for delivery via cell phone messaging and chat applications like WhatsApp or Facebook.

In response to logistics concerns and the midst of the global COVID-19 pandemic, social support programming for this community could best be met not through traditional in-person group meetings but rather through mobile technology. Many of the women in the study sought information on parenting through the internet or their phones. Websites, blogs, WhatsApp groups, and phone communication with families on the mainland were often used to gather information on child development, illness, and emotional support, suggesting that mobile technology may be an essential resource for an intervention. Further, studies in many countries, including Ecuador, have found that distance-based (web, smartphone, or social networking) psychoeducational and social support for women have demonstrated improvements in social support (Shorey et al., 2017), depression (Jiao et al., 2019; Nolan et al., 2018), breastfeeding (Lau et al., 2016), and infant health (Maslowsky et al., 2016). Nurses have been critical in leading many of these interventions, guiding programs like Goal Mama, a maternal and infant health intervention in the United States (Ly et al., 2019); a mobile phone-based postnatal patient support intervention in Ecuador (Maslowsky et al., 2016); and a maternal depression intervention in Australia (Sawyer et al., 2019).

The proposed program has the potential to provide the expert-level education that mothers seek on self-care and child health and development, improve mothers’ social ties, and offer a safe space for women to discuss their concerns with experts and peers. Together, these components may fulfill the needs described by the population, empower women with knowledge and skills to care for their own emotional needs, increase confidence in parenting, improve maternal and child health, and ultimately build up the community on the Galápagos.

## Conclusions

Many researchers have reported high maternal stress and depression on the Galápagos Islands. However, this study is the first to build on that work to consider the best ways to address these needs in alignment with the community’s stated goals and context-specific challenges.

Nonetheless, this study is limited by several important factors. First, our small sample size may limit our understanding of the current challenges that women with young children face. However, our methods sought the diverse perspectives mothers, healthcare workers, and community leaders, and through these interviews, we reached data saturation. Second, we may be limited by our positionality as outsiders ourselves. Nonetheless, the researchers in our group has a long history of health research in the Galápagos Islands. The researchers made a concerted effort to be reflexive, be mindful of power dynamics, and build trust with this community. Further, the methods of SORA are specifically designed to identify this bias, and we used member checking to confirm our findings and themes. Last, while the solutions recommended here may not be generalizable to mainland Ecuador, they may be generalizable to other islands in the Galápagos and potentially to other remote communities.

Despite these weaknesses, this work has many strengths. First, our team has been researching health and developing relationships in the Galápagos for over 10 years, allowing us to interview central community leaders, healthcare workers, and mothers across San Cristóbal. Here, we synthesize these diverse perspectives to streamline potential interventions. Further, our use of SORA allows a nuanced analysis of interview transcripts that maintains the contextual integrity of each interview. Last, this work draws on both participant responses and the current realities of the COVID-19 pandemic to recommend a web-based, mobile technology intervention that is culturally appropriate, feasible, and sustainable for mothers in this remote context.

This research is important for informing a potential intervention program that could improve the lives of mothers of young children on the Galápagos. In considering possible interventions on maternal and child health, we had initially attempted to generalize solutions from research with similar populations from mainland Ecuador and migrant populations elsewhere in the Americas. We hypothesized that home visitation or group programs led by healthcare workers would effectively support mothers of young children on the Galápagos. After examining potential solutions with our participants, however, we found that women’s needs were nuanced and specific to the local context. Ultimately, this work required that we incorporate the voices of women and community members to propose a web-based social support and education intervention led by nurses to address the community’s specific needs. This intervention could address the needs of mothers by improving trust between mothers and with the local hospital, providing information and services on maternal and infant health, and allowing women a safe space to speak freely and develop friendships with other mothers.
